# Study of the Probiotic Potential of Lactic Acid Bacteria Isolated from Artisanal Colonial Cheese and Evaluation of Microencapsulation as a Protective Method Under Simulated Gastrointestinal Conditions

**DOI:** 10.3390/foods15030547

**Published:** 2026-02-04

**Authors:** Ivan De Marco, Vanessa Cortina Zanetti, Ana Paula Zapelini de Melo, Natália Regina Coldebella Ferreira, Callebe Camelo-Silva, Jamile Caroline Siewerdt Duarte Silveira, Mariane Wolf, Silvani Verruck

**Affiliations:** 1 Department of Food Science and Technology, Agricultural Sciences Center, Federal University of Santa Catarina, Florianópolis 88034-001, Santa Catarina, Brazil; 2 Department of Chemical Engineering and Food Engineering, Federal University of Santa Catarina, Florianópolis 88040-970, Santa Catarina, Brazil; 3 Department of Food Engineering and Chemical Engineering, Santa Catarina State University, Pinhalzinho 89870-000, Santa Catarina, Brazil

**Keywords:** Brazilian cheeses, probiotic isolation, spray-drying microencapsulation, cell viability

## Abstract

Artisanal colonial cheese (ACC) produced from raw milk is a rich reservoir of autochthonous lactic acid bacteria (LAB), but strain-level evidence supporting safe downstream application and technological stability remains limited. In this study, 10 LAB isolates from ACC were screened for phenotypic safety, antimicrobial susceptibility, and probiotic-related traits, and their viability was further assessed after inulin-based spray-drying microencapsulation under different storage temperatures. All isolates showed no hemolytic or mucinolytic activity and did not produce gelatinase, supporting an initial safety profile, and all strains were sensitive to at least two antimicrobial classes. Strain prioritization identified *Lacticaseibacillus casei* LAB06, LAB09, and LAB10 and *Lactiplantibacillus plantarum* LAB03 as the most robust candidates for downstream development because they maintained stable cell counts throughout simulated gastrointestinal digestion. Inulin spray-drying yielded structurally stable microcapsules and supported refrigerated storage, with substantially lower viability losses at 4 °C than at 25 °C; notably, *L. plantarum* LAB01 and LAB02 showed the best refrigerated shelf-life, remaining above 6.0 log CFU/g after 45 days. Together, these results position ACC as a source of promising LAB candidates and highlight cold-chain-compatible microencapsulation as a strategy to support safe functional food development with potential public health benefits.

## 1. Introduction

Artisanal Colonial Cheese (ACC) is a traditional product from southern Brazil, with historical roots linked to European colonization, particularly by Portuguese, Italian, and German immigrants. Produced from raw bovine milk, ACC exhibits distinctive sensory characteristics, such as a soft texture and yellowish color, which evolve throughout the maturation process. This period typically ranges from 15 to 30 days and is defined by the producer [1,2,3]. Like other artisanal cheeses, ACC reflects local environmental conditions and traditional know-how, serving as a rich source of microbial diversity with implications for both product quality and functional potential.

The microbiota of raw milk directly influences the final profile of the cheese, driving complex biochemical reactions during maturation, such as proteolysis, lipolysis, and carbohydrate fermentation, which lead to the formation of volatile compounds, organic acids, and fatty acids [4,5]. Among the microorganisms present, lactic acid bacteria (LAB) play a key role by contributing to fermentation and inhibiting undesirable microbes. Certain LAB strains, such as *Lacticaseibacillus casei*, *Lactiplantibacillus plantarum*, *Levilactobacillus brevis*, and *Pediococcus acidilactici*, display functional traits consistent with probiotic potential, provided they meet criteria such as resistance to the gastrointestinal tract, ability to adhere to the intestinal epithelium, absence of virulence factors, and susceptibility to antimicrobials [6,7,8,9].

However, the viability of probiotic strains can be compromised during food processing and storage. Factors such as temperature, humidity, and oxygen negatively affect their stability [10]. In this context, microencapsulation by spray drying emerges as an effective strategy to protect these bacteria, enhancing their resistance to adverse conditions and improving their release in the intestinal tract [11]. Additionally, the use of prebiotic carrier agents, such as inulin, can potentiate probiotic efficacy through functional synergy [12].

Therefore, the aim of this study was to isolate and characterize LAB from ACC produced in the western region of Santa Catarina, Brazil, and to evaluate their probiotic potential for the first time through phenotypic safety assessment and functional characterization. Additionally, the study investigated the effect of spray-drying microencapsulation using inulin as a carrier on the viability and structural stability of selected LAB strains during storage.

## 2. Materials and Methods

### 2.1. Obtaining ACC Samples

ACC samples were produced using raw milk and a 21-day maturation period in two rural dairies located in Seara, SC, Brazil (27°08′58″ S, 52°18′38″ W). Considering the microbiological dynamics during maturation, sampling was carried out on days 14 and 21, a period characterized by greater selectivity for LAB and a reduction in non-lactic acid bacteria. Sampling was performed at the production sites, and the cheeses were transported under refrigeration (<7 °C) in insulated boxes and in their original packaging to the laboratory, where they were stored at −18 °C until analysis.

### 2.2. Isolation and Morphological Characterization of Potentially Probiotic LAB by SEM

LAB were isolated on Man Rogosa Sharpe agar (MRS) (Sigma-Aldrich, St. Louis, MO, USA) using the pour plate technique with an overlay, according to Tanaka et al. [13]. Briefly, 25 g aliquots of ACC were added to 225 mL of 0.85% (*w*/*v*) saline solution and homogenized in a sample homogenizer (model MA 440/CF, Marconi, São Paulo, Brazil) for 2 min. From the initial dilution (10^−1^), serial dilutions were prepared, plated on MRS agar, and incubated for 72 h at 37 °C under anaerobic conditions (Anaerocult, Merck, Darmstadt, Hesse, Germany). Isolates exhibiting typical LAB characteristics (Gram-positive, rod- or cocci-shaped and catalase-negative) were selected and stored at −20 °C in MRS broth supplemented with 20% (*v*/*v*) glycerol until further analysis.

Morphological analysis of the isolated strains was performed according to Silva et al. [14]. Cultures were grown in MRS broth for 24 h at 37 °C, washed twice with 0.85% (*w*/*v*) saline solution, and centrifuged at 4000× *g* for 10 min. The resulting pellets were frozen at −20 °C for 24 h, followed by freezer-drying. Subsequently, samples were individually mounted on stubs with carbon tape and coated with a thin layer of gold by vacuum sputtering (Leica EM SCD 500, Wetzlar, Germany). Cell morphology was examined using a scanning electron microscope (JSM 6390 LV, Jeol, Tokyo, Japan) under an accelerating voltage of 8 kV, at magnifications of 6000×, 7500×, and 10,000×, and working distances of 1–2 µm.

### 2.3. Genotypic Identification

Bacterial identification was performed by high-throughput sequencing of the V3/V4 regions of the 16S rRNA gene. Library preparation followed a proprietary protocol (Neoprospecta Microbiome Technologies, Florianópolis, SC, Brazil). Amplification was carried out using primers targeting the V3–V4 region of the 16S rRNA gene: 341F (CCTACGGGRSGCAGCAG) [15] and 806R (GGACTACHVGGGTWTCTAAT) [16].

Libraries were sequenced on the MiSeq Sequencing System (Illumina Inc., San Diego, CA, USA) using paired-end reads with the MiSeq Reagent Kit v3 (600 cycles). Sequence data were processed using the Sentinel pipeline (Neoprospecta Microbiome Technologies, Florianópolis, SC, Brazil), following established bioinformatic approaches described by Altschul et al. [17], Andrews [18], Cock et al. [19] and Masella et al. [20]. The obtained DNA sequences were compared against the Sentinel database [21] and the Greengenes database [22].

### 2.4. Hemolytic Activity

The isolated strains were streaked onto tryptic soy agar supplemented with 5% sheep blood (LaborClin, Pinhais, Brazil) and incubated at 37 °C for 48 h, as described by Margalho et al. [23]. Hemolytic activity was assessed based on halo formation around the colonies and classified as follows: greenish halos, indicating partial hemolysis (α-hemolysis); absence of halos, corresponding to non-hemolysis (γ-hemolysis); and transparent or clear halos, indicating complete hemolysis (β-hemolysis). *Staphylococcus aureus* ATCC 25923 was used as the positive control for β-hemolysis.

### 2.5. Gelatinase Production

Aliquots of 10 µL of each isolated strain were inoculated onto Luria–Bertani agar supplemented with 3% gelatin (*w*/*v*). The plates were incubated at 37 °C for 48 h and subsequently kept at 4 °C for 4 h. Gelatin hydrolysis was determined by the formation of halos around the colonies. *S. aureus* ATCC 25923 was used as the positive control for gelatinase production [24].

### 2.6. Mucin Degradation Capacity

The isolated strains were inoculated on MRS agar and modified MRS-M agar (supplemented with 3% (*w*/*v*) glucose), both containing 0.5% (*w*/*v*) porcine gastric mucin (Type III, M1778, Sigma-Aldrich, St. Louis, MO, USA), and incubated at 37 °C for 72 h. For result interpretation, the plates were stained with 2% (*w*/*v*) malachite green in 3.5 M acetic acid, according to Leska et al. [25]. *Salmonella typhimurium* ATCC 14028 was used as the positive control.

### 2.7. Antimicrobial Susceptibility Testing

Antimicrobial susceptibility assays were performed according to ISO 20776-1 [26] using the minimum inhibitory concentration (MIC) method. Antibiotic dilutions (ampicillin, benzylpenicillin, cephalexin, clindamycin, metronidazole, meropenem, and streptomycin) were prepared to obtain concentrations above and below the established cutoff values for each compound. Cutoff values were defined based on the European Commission (EUC) [27], the European Food Safety Authority (EFSA) [28], the European Committee on Antimicrobial Susceptibility Testing (EUCAST) [29], and the study by Gad et al. [30].

Inocula were grown in MRS broth for 16 h at 37 °C and adjusted in a spectrophotometer (K37-VIS, KASVI, Pinhais, PR, Brazil) at 625 nm to an absorbance range of 0.08–0.13. Subsequently, 10 μL of the standardized inoculum was added to each well, and the microplates were incubated at 37 °C for 24 h. Microbial growth was confirmed by the addition of 20 μL of a 0.5% (*w*/*v*) 2,3,5-triphenyltetrazolium chloride (TTC) solution, followed by incubation at 37 °C for 1 h. The LAB strains *L. plantarum* ATCC 8014 and *L. casei* BGP 93 were used as controls. The MIC was defined as the lowest antimicrobial concentration that inhibited visible microbial growth, indicated by the absence of color development.

The minimum bactericidal concentration (MBC) was determined according to Celiktas et al. [31], with modifications. Aliquots (10 µL) from wells showing no visible growth (i.e., concentrations at or below the MIC) were surface-inoculated onto MRS agar and incubated at 37 °C for 24 h. The lowest antimicrobial concentration at which no microbial growth was observed on the agar plates was defined as the MBC.

### 2.8. Evaluation of LAB Survival in the Gastrointestinal System In Vitro

Survival assays of LAB isolates under simulated gastrointestinal conditions were conducted according to the INFOGEST protocol proposed by Brodkorb et al. [32], with adaptations. pH adjustments were performed using 0.1 M NaOH or 0.1 M HCl, as required. The temperature was maintained at 37 °C, and peristaltic movements were simulated using a Dubnoff digital water bath (Model NI 1232, Nova Instruments, Piracicaba, SP, Brazil) [33]. Enzymatic solutions were prepared in advance and sterilized by membrane filtration through a 0.22 µm filter (MF-Millipore, Billerica, MA, USA). After sterilization, all solutions were kept in an ice bath until use.

Initially, 1 mL of bacterial cultures, presenting an approximate concentration of 8.0 log CFU/mL based on the 0.5 McFarland standard, was transferred to sterile test tubes. Simulated gastrointestinal digestion was performed in two sequential stages, corresponding to the gastric and intestinal phases. For the gastric phase, the oral bolus was mixed with simulated gastric fluid (SGF) at a 1:1 ratio, followed by the addition of CaCl_2_ to a final concentration of 0.15 mM. Pepsin was then added to achieve an activity of 2000 U/mL, and the mixture was incubated under agitation for 2 h at 37 °C, with the pH adjusted to 3.0. Subsequently, the intestinal phase was initiated by mixing the gastric bolus with simulated intestinal fluid (SIF) at a 1:1 ratio. Bile salts (10 mM), CaCl_2_ (0.6 mM), and pancreatin were added, considering a final trypsin activity of 100 U/mL. Incubation was carried out under agitation for 2 h at 37 °C, with the pH adjusted to 7.0.

After each digestion stage, samples were subjected to viable cell counting. Aliquots were inoculated in duplicate by the pour plate method with overlay on MRS agar and incubated at 37 °C for 72 h. Colonies were counted, and results were expressed as log CFU/mL. The bacterial survival rate (%) was calculated as described by [34].

### 2.9. Auto-Aggregation and Hydrophobicity Test

The isolated strains were cultured in MRS broth for 18 h at 37 °C, centrifuged (4000 rpm, 10 min), washed twice, and resuspended in phosphate-buffered saline (PBS; pH 7.4) until an optical density of 0.5 at 600 nm (A_0_) was reached. Aliquots of 2 mL of each suspension were incubated at 37 °C for 3, 6, and 24 h. After each incubation period, the absorbance was measured (A_t_). The percentage of auto-aggregation was calculated based on the absorbance variation over time, according to Yasmin et al. [35].

Hydrophobicity was determined by bacterial adhesion to hydrocarbons (BATH), according to Farid et al. [36]. The strains were cultured in MRS broth for 24 h at 37 °C, centrifuged (4000× *g*, 15 min), washed, and resuspended in phosphate-buffered saline (PBS; pH 7.4) to obtain an initial optical density (OD) of 0.7 at 600 nm. Subsequently, 3 mL of the bacterial suspension was mixed with 1 mL of dichloromethane, incubated at 37 °C for 10 min, vortexed for 30 s, and then incubated for an additional 1 h to allow phase separation. The absorbance of the aqueous phase (ODfinal) was measured, and cell hydrophobicity was calculated based on the reduction in absorbance.

### 2.10. Microencapsulation of LAB Strains

Previously activated cultures were inoculated at 1% (*v*/*v*) into MRS broth and incubated at 37 °C for 24 h. After incubation, the cells were centrifuged at 4000× *g* for 10 min and washed with sterile saline solution (0.85%, *w*/*v*), followed by a second centrifugation under the same conditions. The resulting pellets were resuspended in 5 mL of sterile saline solution (0.85%, *w*/*v*) under gentle agitation. The cell suspension was incorporated into the carrier agent, inulin (200 g L^−1^), previously dissolved in sterile natural mineral water. The mixture was maintained under constant magnetic stirring to ensure homogeneity prior to and atomization in a spray dryer (B-290, Buchi, Switzerland), operating with an inlet temperature of 150 °C, outlet temperature of 50 ± 3 °C, feed rate of 20 mL min^−1^, air flow of 35 m^3^ h^−1^, and pressure of 0.7 MPa. The resulting powders were collected in sterile Falcon tubes and stored at −20 ± 1 °C until further analyses.

### 2.11. Survival of LAB After Spray Drying and During Shelf-Life

The viability of LAB strains was evaluated before microencapsulation (feeding solution), immediately after spray drying, and during storage at 25 ± 1 °C and 4 °C for 15, 30, 45, and 60 days. For the analyses, 1 g of each sample was aseptically transferred to 9 mL of sterile phosphate buffer (pH 7.0; 0.1 mol L^−1^) and homogenized for 5 min in a sample homogenizer to ensure complete release of the encapsulated cells. From the initial dilution (10^−1^), serial dilutions were prepared and plated in triplicate on MRS agar. The plates were incubated at 37 °C for 72 h. Viability was expressed as log CFU/mL, and the bacterial survival rate (%) was calculated as previously described.

### 2.12. Morphology and Size of Microparticles

The morphology and size of the microparticles were analyzed by SEM at an accelerating voltage of 10 kV with magnifications ranging from 300 to 4000×. For sample preparation, the microparticles were mounted on stubs using carbon tape and coated with a thin layer of gold. The particle diameter was estimated from the micrographs at their original magnification using ImageJ software (version 1.54g).

### 2.13. Interaction Between Microcapsule Components by FTIR

The interaction between the constituent components of the microcapsules was evaluated by Fourier-transform infrared spectroscopy (FTIR) using an IRPrestige-21 spectrophotometer (Shimadzu Scientific Instruments Inc., Columbia, MA, USA). Samples were prepared in pellet form using potassium bromide (KBr) as the dispersing matrix. Spectral analyses were performed in the scanning range of 550 to 4000 cm^−1^ with a resolution of 4 cm^−1^ [34].

### 2.14. Thermogravimetric Analysis

Thermogravimetric analysis (TGA) and derivative thermogravimetry (DTG) were carried out using an STA 449 F3 Jupiter thermal analyzer (Netzsch, Selb, DE, Germany). For each analysis, approximately 6.5 mg of microcapsules or inulin was placed in aluminum pans and heated from 30 to 300 °C at a heating rate of 10 °C/min. Measurements were conducted under an inert nitrogen atmosphere with a controlled flow rate of 20 mL/min.

### 2.15. X-Ray Diffraction (XRD)

The crystalline properties of the microcapsules and inulin were determined by X-ray diffraction using a Rigaku MiniFlex600 diffractometer (Rigaku Corporation, Tokyo, Japan). Analyses were performed over a range of 5 to 60°, with a scanning step of 0.05°/s and a scanning speed of 10°/min.

### 2.16. Statistical Analyses

Data were expressed as mean ± standard deviation. Statistical analyses were performed using Statistica^®^ 14.0.1 software (TIBCO Software Inc., Palo Alto, CA, USA). Triplicate data were subjected to analysis of variance (ANOVA) followed by Tukey’s test (5% significance level).

## 3. Results and Discussion

### 3.1. Identification of LAB Strains

**Figure 1:**
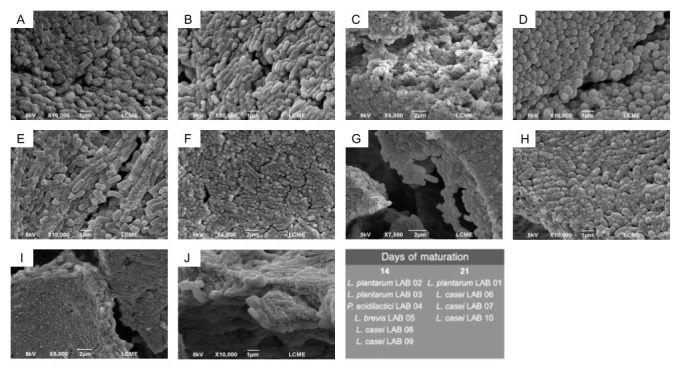
Morphology of LAB strains with probiotic potential isolated from ACC at 14 and 21 days of maturation. (**A**) *L. plantarum* LAB01. (**B**) *L. plantarum* LAB02. (**C**) *L. plantarum* LAB03. (**D**) *P. acidilactici* LAB04. (**E**) *L. brevis* LAB05. (**F**) *L. casei* LAB06. (**G**) *L. casei* LAB07. (**H**) *L. casei* LAB08. (**I**) *L. casei* LAB09. (**J**) *L. casei* LAB10.

LAB were isolated at two distinct maturation stages of ACC, at 14 and 21 days, reflecting different phases of the product’s microbial dynamics. All ten isolates exhibited typical LAB characteristics, being Gram-positive and catalase-negative. Morphological analysis by SEM (Figure 1) revealed a predominance of rod-shaped cells (*n* = 9) and one strain with coccoid morphology (n = 1). Overall, bacterial cells showed localized signs of cell wall damage, possibly associated with the absence of protective agents during culture lyophilization for morphological analysis. However, the overall structural integrity and cellular morphology were preserved, indicating that these alterations did not compromise strain identification or initial characterization.

Genotypic identification confirmed the presence of *L. plantarum* (n = 3), *P. acidilactici* (n = 1), *L. brevis* (n = 1), and *L. casei* (n = 5). The strains were designated sequentially as LAB followed by a cardinal number. Among the isolates obtained after 14 days of maturation, the strains were identified as *L. plantarum* LAB02 and LAB03, *P. acidilactici* LAB04, *L. brevis* LAB05, and *L. casei* LAB08 and LAB09. In contrast, strains isolated after 21 days of maturation corresponded to *L. plantarum* LAB01 and *L. casei* LAB06, LAB07, and LAB10 (Figure 1). The only strain exhibiting coccoid morphology was confirmed as *P. acidilactici* LAB04 (Figure 1D), which displayed the characteristic paired-cell arrangement of this genus.

The predominance of species belonging to the genus *Lactobacillus* is consistent with their traditional use as starter or adjunct cultures in the production of a wide range of fermented foods, such as cheeses and yogurts, mainly due to their well-established GRAS status [37]. Species such as *L. casei*, *L. plantarum*, and *L. paracasei* are frequently reported as dominant during cheese maturation, whereas *L. brevis* is more commonly associated with the later stages of the maturation process [38].

Similar results have been reported in studies involving traditional dairy products. Hadef et al. [39], in evaluating different traditional dairy products from Algeria, including cheeses, identified 25 LAB strains, mainly belonging to the genera *Lactobacillus*, *Lactococcus*, *Enterococcus*, and *Leuconostoc*, with *Lactobacillus* predominant in cheese samples. Dosuky et al. [40] reported that, among 74 LAB strains isolated from salted cheese whey, all characterized as Gram-positive and catalase-negative, 13 isolates (17.6%) belonged to the genus *Lactobacillus*. These findings corroborate the results of the present study and reinforce ACC as a relevant source of LAB with probiotic potential and applicability in functional food development.

### 3.2. Safety Analyses of LAB Isolates

The LAB isolates evaluated showed no hemolytic or mucinolytic activity and did not produce gelatinase, indicating the absence of virulence factors associated with host invasion. Hemolysis is related to the destruction of red blood cells [41], gelatinase activity to the degradation of structural components such as collagen and elastin [42], and mucinase activity to the breakdown of mucin, a key protective barrier of the intestinal epithelium [43].

The susceptibility of the isolated LAB strains to seven antibiotics commonly used in clinical and hospital settings is presented in Table 1. All antibiotics tested showed at least one resistant strain; however, all isolates were sensitive to at least two different classes of antibiotics. Castro-López et al. [44] reported that antimicrobial resistance is species-dependent and may vary even among strains of the same species. The EFSA [28] document does not provide a breakpoint value for *L. plantarum* against streptomycin; therefore, the cutoff established by the EUC [27] was used. Based on this criterion, among the three *L. plantarum* isolates, LAB02 and LAB03 were classified as resistant.

Streptomycin resistance in *L. plantarum* is considered intrinsic, as the uptake of this antibiotic depends on cytochrome-mediated electron transport, a system absent in *Lactobacillaceae*, rendering these bacteria unable to internalize the compound [42]. Anisimova and Yarullina [45] also reported streptomycin resistance in 12 *L. plantarum* strains. For the remaining strains evaluated in the present study, streptomycin sensitivity was observed.

Li et al. [46] reported that several species within the *Lactobacillaceae* family exhibit resistance to antibiotics that inhibit synthesis of nucleic acid or cell wall, such as metronidazole, benzylpenicillin, and cephalexin. In the present study, all LAB strains in the present study were resistant to these antimicrobials, with the exception of *L. casei* LAB08, which was sensitive to benzylpenicillin. However, resistance to ampicillin, also a cell wall synthesis inhibitor, was observed only in *L. plantarum* LAB03 and *L. casei* LAB08. This resistance may have developed due to selective pressure in the isolation environment and may involve transferable resistance genes, such as those encoding extended-spectrum β-lactamases (ESBLs), including *blaZ* and *blaSHV* [42,46].

Within the carbapenem class, two *L. casei* isolates (LAB07 and LAB10) exhibited resistance to meropenem. DeMarco et al. [47] identified meropenem-resistant *Lactobacillaceae* in an immunocompromised patient consuming probiotics, with *L. casei* among the isolates. Anisimova et al. [48] also reported meropenem-resistant *Lacticaseibacillus paracasei* strains originating from probiotic supplements. In contrast, Duche et al. [49] did not detect imipenem resistance among probiotic bacteria; however, they reported meropenem resistance in isolates of *L. brevis, L. paracasei, Lactiplantibacillus pentosus*, and *L. casei.* These authors suggested that carbapenem resistance in *Lactobacillaceae* may be species-specific, which is consistent with the findings of the present study.

Nevertheless, studies investigating the susceptibility of probiotic microorganisms to meropenem remain scarce, and information on resistance patterns is still limited. Meropenem resistance is believed to be intrinsic, as this antibiotic belongs to the β-lactam class and acts by inhibiting cell wall synthesis. *Lactobacillaceae* are generally sensitive to antimicrobials that inhibit protein synthesis, such as clindamycin [50]. In this study, only the *L. brevis* LAB05 strain exhibited resistance to clindamycin. Silva et al. [51], when evaluating probiotic LAB isolated from artisanal Minas cheese, reported *L. casei* and *L. plantarum* strains to be clindamycin-sensitive while also identifying clindamycin-resistant *L. brevis* isolates.

The MBC results showed no bactericidal activity for antibiotics that exhibited 100% resistance (cephalexin and metronidazole) within the concentration range evaluated (Table 1). Benzylpenicillin, which showed resistance in most isolates, presented MBC values close to the upper limit of the tested range. Some antibiotics exhibited identical MIC and MBC values; however, for others MBC values were not observed within the evaluated range, even when the corresponding strains were classified as sensitive (Table 1). The safety-related results obtained in this study provide an important first-line screening of the LAB isolates, indicating no phenotypic evidence of antimicrobial resistance or virulence-associated traits. These findings support the selection of candidate strains from a large pool of isolates and suggest a favorable safety profile. However, the choice of strains for subsequent in vivo testing should be supported by complementary molecular analyses to confirm the absence of resistance and virulence genes and to strengthen the safety assessment for functional applications.

### 3.3. Survival in the In Vitro Gastrointestinal System

To exert beneficial health effects, probiotics must reach the intestine at high concentrations and withstand the adverse conditions of the gastrointestinal tract [52]. As shown in Table 2, the evaluated LAB strains exhibited good tolerance to the simulated gastrointestinal system, maintaining counts above 7.0 log CFU/g even under low pH conditions and in the presence of bile salts. Among the five *L. casei* strains evaluated, three (LAB06, LAB09, and LAB10) maintained stable cell counts throughout the in vitro digestion process, with no significant reductions (*p* > 0.05). Among the *L. plantarum* strains, only LAB03 maintained stable cell counts throughout the simulated digestion process. In contrast, *L. plantarum* LAB01 showed reduction (*p* < 0.05) under intestinal conditions, while LAB02 exhibited decreased counts at all simulated digestion stages. Both *L. brevis* LAB05 and *P. acidilactici* LAB04 exhibited significant reductions (*p* < 0.05) after the gastric phase, but only *L. brevis* LAB05 showed a reduced survival rate. Although a reduction in viable counts was observed after simulated digestion, all strains maintained cell counts above 6.0 log CFU/g, which is the minimum threshold required to exert probiotic effects [53]. In addition, rapid recovery following gastric stress is considered essential for intestinal viability [54].

The resistance of LAB has been associated with innate and adaptive defense mechanisms involving coordinated physiological and metabolic responses that enable cells to cope with acidic pH, digestive enzymes, and bile salts. These defense strategies are strongly strain-dependent and influenced by environmental conditions. Among the most relevant mechanisms are the ability to sense environmental stress and activate signaling and export systems, which are essential for maintaining cellular homeostasis. The accumulation of compatible solutes allows cells to adjust internal osmotic pressure, supporting growth and cell division under adverse conditions. In addition, modulation of energy production enables the reorganization of intracellular carbon reserves, while metabolic adaptation through the redirection of biochemical pathways and the use of alternative energy sources helps sustain cellular metabolism during stress. Structural adaptation of the cell envelope, including modifications in membrane and cell wall composition, contributes to cell stability and reduces the impact of external stressors. Furthermore, the synthesis of stress-related proteins, such as chaperones and proteases, plays a key role in protecting and repairing damaged macromolecules. Finally, the production of antimicrobial compounds may provide a competitive advantage and support persistence in complex microbial environments [55,56].

This tolerance can also be improved by the production of bile salt hydrolase (BSH) by some probiotics [57]. Ait Chait et al. [52] similarly reported strain-specific variability among *L. brevis* isolates from artisanal cheeses, with reductions below 1.0 log CFU/g. Margalho et al. [58] demonstrated that *L. plantarum* and *P. acidilactici* strains isolated from ACC maintained survival rates between 75 and 100% in the presence of 0.4% bile salts and at pH values ranging from 2.5 to 3.5, conditions comparable to those applied in the present study. According to these authors, approximately 25% of strains isolated from Brazilian artisanal cheeses exhibited bile salt resistance, which was influenced by the microbial profile of the isolation source. Furthermore, the isolated strains naturally display greater tolerance to low pH due to the acidic nature of the ACC matrix from which they originate, as well as the production of organic acids by LAB, which requires increased tolerance for survival within the matrix. This contributes to their viability under gastric conditions and to the low losses observed during digestion [58].

### 3.4. Auto-Aggregation and Hydrophobicity Assay

Auto-aggregation is a key criterion in probiotic selection, as it is associated with gastrointestinal tract colonization and antagonism activity against pathogens [56]. All LAB strains analyzed exhibited good auto-aggregation capacity after 24 h, with higher values observed for *L. brevis* LAB05 and *L. casei* LAB08 (Table 3). With the exception of *L. casei* LAB08, which showed no significant variation in auto-aggregation over time (*p* > 0.05), the remaining strains exhibited a time-dependent increase in aggregation. These results are consistent with previous studies evaluating LAB strains isolated from artisanal cheeses [52,59].

Bacterial auto-aggregation is influenced by several factors, including cell surface charge and composition, which may involve components released during autolysis, such as polysaccharides and extracellular DNA, as well as the presence of aggregation-promoting genes. Strains exhibiting aggregation rates above 50% are frequently reported to express this phenotype more efficiently, reinforcing their potential probiotic functionality [59,60].

Cell surface hydrophobicity is associated with the adhesion of probiotic bacteria to the intestinal epithelium [58] and depends on components such as proteins, polysaccharides, and lipoteichoic acids [60]. In Table 3, the results showed high hydrophobicity levels, particularly for *L. brevis* LAB05 and *L. casei* LAB07. The strains *L. plantarum* LAB01, LAB02, and LAB03 exhibited significant variations (*p* < 0.05), with values below 90%, while *P. acidilactici* LAB 04 presented the lowest index (<50%). Among the *L. casei* isolates, three strains (LAB06, LAB07, and LAB10) displayed hydrophobicity above 90%, a behavior also reported by Ait Chait et al. [52]. According to Barzegar et al. [61] and Guan et al. [62], these results reflect the strain-specific composition of the cell wall, which may include intercalated proteins, hydrophobic amino acids, lipids, and other factors such as the bacterial growth phase, all of which can influence hydrophobicity.

### 3.5. Survival of LAB After Spray-Drying and During Shelf-Life

Several mechanisms have been proposed to explain the action of protective agents during the drying process, particularly with regard to the maintenance of bacterial viability immediately after drying and throughout storage. However, as highlighted by Van Engeland et al. [63], these mechanisms have not yet been fully elucidated. Among the main mechanisms described in the literature are modifications in cell membrane fluidity, the accumulation of compounds involved in osmoregulation, the prevention of oxidative processes, cell coating, increased thermal resistance, the application of prior osmotic dehydration, and changes in drying kinetics.

**Figure 2:**
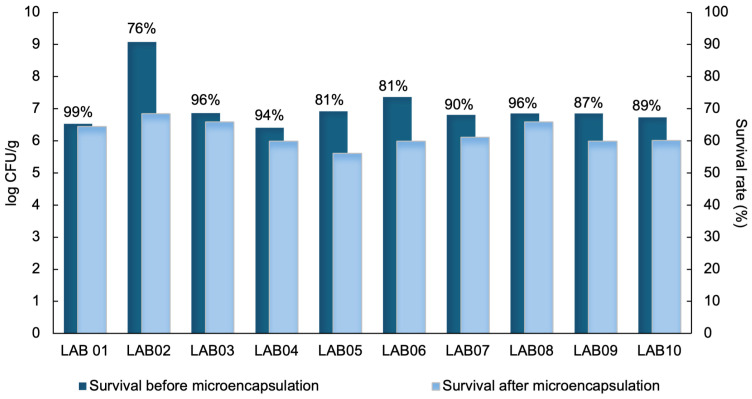
Survival rate of LAB strains *L. plantarum* (LAB01, LAB02, and LAB03), *P. acidilactici* (LAB04), *L. brevis* (LAB05), and *L. casei* (LAB06, LAB07, LAB08, LAB09, and LAB10) before and after the microencapsulation process by spray drying.

After the drying process, the powders exhibited an average water activity (a_w_) of 0.27 and a mean moisture content of 4.5%, values considered suitable for product stability, as they limit the occurrence of undesirable biochemical and microbiological reactions [64,65]. Under these conditions, the survival rate of strains microencapsulated by spray drying ranged from 76% to 99% (Figure 2). With the exception of the strain *L. brevis* LAB05, which presented a count of 5.4 log CFU/g, all other strains exhibited viable counts above 6.0 log CFU/g immediately after atomization (Figure 2) and were therefore considered suitable for functional applications [11].

**Figure 3:**
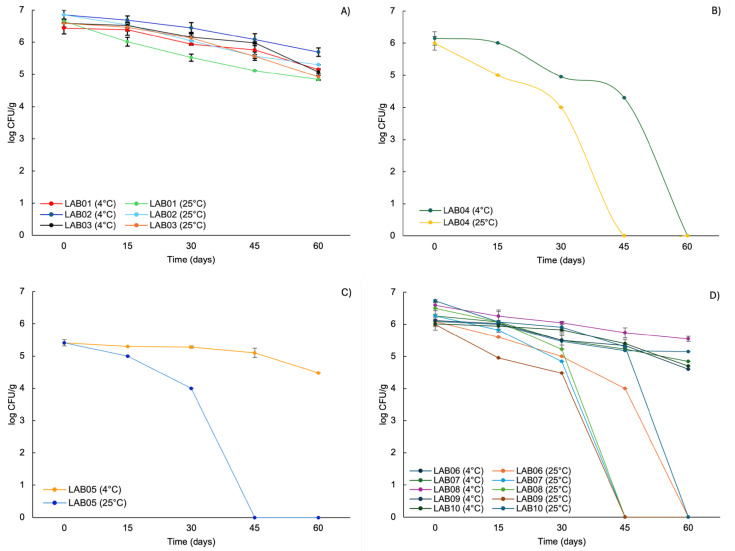
Survival of probiotic microparticles during 60 days of storage at 4 °C and 25 °C, where (**A**): microparticles containing *L. plantarum* (LAB01, LAB02, and LAB03), (**B**): *P. acidilactici* (LAB04), (**C**): *L. brevis* (LAB05), and (**D**): *L. casei* (LAB06, LAB07, LAB08, LAB09, and LAB10). Error bars represent the standard deviation of the experimental mean.

Regarding sensitivity to the microencapsulation process, the strains *L. plantarum* LAB02, *L. brevis* LAB05, and *L. casei* LAB06 showed the greatest reductions in viability, with losses of up to 2.0 log CFU/g (*p* < 0.05) (Figure 2). Interestingly, these same strains demonstrated better performance during storage at 4 °C throughout the evaluated period, with an average reduction of approximately 1.0 log CFU/g after 60 days, suggesting greater stability under refrigerated conditions (Figure 3).

The maintenance of viability above the minimum threshold of 6.0 log CFU/g during the first 15 days of storage at 4 °C was observed for all microencapsulated strains (except *L. brevis* LAB05). After 30 days, only the strains *L. plantarum* LAB02 (6.4 log CFU/g), *L. plantarum* LAB03 (6.1 log CFU/g), and *L. casei* LAB08 (6.0 log CFU/g) maintained counts above this threshold. At 45 days, only *L. plantarum* LAB02 remained above the functional viability limit. Overall, storage at 4 °C proved to be more effective in preserving strain viability throughout storage, whereas more pronounced reductions were observed at 25 °C, with an average loss of 4.8 log CFU/g after 60 days.

Among the evaluated strains, those belonging to the species *L. plantarum* (LAB01, LAB02, and LAB03) exhibited greater resistance during storage at both temperatures, reaching the end of the experimental period with counts close to 5.0 log CFU/g (Figure 3A). In contrast, the strain *P. acidilactici* LAB04 showed significant losses in viability (*p* < 0.05) when stored at both 4 °C and 25 °C (Figure 3B), with reductions of 1.8 and 5.9 log CFU/g after 45 days, respectively. After 60 days of storage, this strain was no longer detected (<1.0 log CFU/g) at either temperature evaluated.

These results support the hypothesis that storage at low temperature (4 °C) minimizes chemical reactions and interactions associated with cellular degradation. The higher viability observed in strains stored under refrigeration indicates that reduced temperatures contribute to the preservation of probiotic structural integrity by delaying mechanisms that compromise their survival [66]. The reductions in viability observed in this study may be related to the lower intrinsic heat resistance of certain strains, as well as to their limited ability to adapt to the thermal, osmotic, oxidative, and mechanical stresses imposed during spray drying microencapsulation [67]. These stresses affect membrane lipids and promote the loss of functional protein conformation, resulting in irreversible damage to bacterial cells [64].

Another possible explanation for the lower viability compared to that reported in some studies in the literature is related to the concentration of inulin used as a wall material. According to Bazzaz et al. [68], inulin concentrations above 20% may compromise LAB viability due to disruption of the polymeric network formed during encapsulation. In addition, the authors highlight that vigorous agitation of the encapsulating solution may lead to destruction of the protective capsule, thereby reducing process efficiency.

Inulin is a non-digestible carbohydrate widely recognized for its prebiotic function and is frequently used as a wall material in microencapsulation processes [11]. However, the results of the present study, together with evidence from the literature, indicate that the association of inulin with other wall materials may promote greater preservation of cell viability. This effect has been attributed to modifications in the physicochemical properties of the microcapsules, particularly an increase in glass transition temperature (Tg), which enhances the protective effect of the encapsulating matrix [69,70].

In this context, Russo et al. [71] evaluated the encapsulation of *Lactobacillus* strains using a combination of alginate (1%), inulin (15%), and maltodextrin (10%) and observed that the spray drying process did not promote significant reductions in viability, with counts above 8.0 log CFU/g after microencapsulation. During storage at 4 °C, viability varied among strains, with a gradual reduction over time, remaining between 6.5 and 8.5 log CFU/g after 60 days. Similarly, Kumar et al. [72], when comparing maltodextrin (20%), inulin (20%), and their combination (10% + 10%) as wall materials, reported greater survival of *L. plantarum* MTCC 25432 when the combination was used (7.4 log CFU/g). Nevertheless, inulin alone showed better performance than maltodextrin, with counts of 7.1 and 6.0 log CFU/g, respectively. Considering the operational conditions adopted in these studies, particularly the inlet temperatures of 130 °C [71] and 120 °C [72], the lower viability observed in the present study may be at least partly related to the use of a higher inlet temperature (150 °C), which tends to intensify the thermal stresses imposed on cells during spray drying microencapsulation.

### 3.6. Morphology and Size of LAB-Containing Microparticles

**Figure 4:**
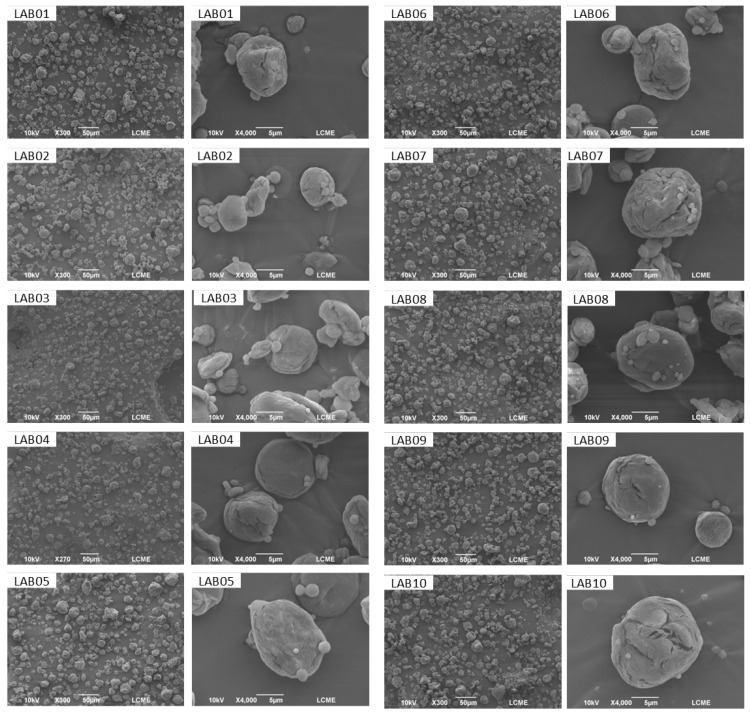
Scanning electron microscopy (SEM) micrographs of microcapsules containing *L. plantarum* (LAB01, LAB02, and LAB03), *P. acidilactici* (LAB04), *L. brevis* (LAB05), and *L. casei* (LAB06, LAB07, LAB08, LAB09, and LAB10) encapsulated with inulin, at magnifications ranging from 300× to 4000×.

The SEM micrographs of the microparticles containing the isolates *L. plantarum* (LAB01–03), *P. acidilactici* (LAB04), *L. brevis* (LAB05), and *L. casei* (LAB06–10), encapsulated with inulin, revealed predominantly spherical particles featuring fissures and irregular shapes (Figure 4). Similar patterns were reported by Verruck et al. [73], attributed to the slow formation of the crust during spray drying, which induces surface stresses and leads to cracking [74]. Nevertheless, these fissures did not compromise the structural integrity of the microparticles, indicating effective protection of the probiotic cells. The absence of typical bacillus or coccus structures further confirms the efficiency of the encapsulation. However, such fissures may facilitate cellular oxidation, potentially affecting viability during storage [73].

The microparticles exhibited sizes ranging from 1.19 to 5.71 µm, with no significant differences (*p* < 0.05) among the encapsulated strains. Particle size can be influenced by factors such as solution viscosity, atomization parameters, feed rate, and air temperature [75]. More viscous solutions tend to produce larger droplets, resulting in larger particles after drying. To avoid this effect, the solution was kept under continuous stirring until atomization, ensuring homogeneity. The small particle sizes obtained (<40 µm) favor dispersion in food matrices, contributing to improved texture and sensory acceptance [66,76].

### 3.7. Interaction Between Microcapsule Components by FTIR

**Figure 5:**
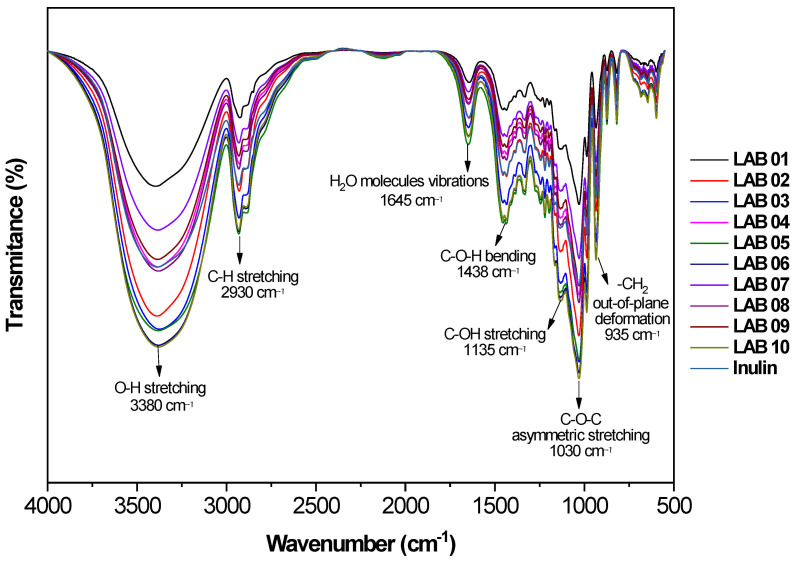
FTIR spectra (4000–550 cm^−1^) of inulin and inulin-based microencapsulated probiotic strains obtained by spray drying: *L. plantarum* (LAB01, LAB02, and LAB03), *P. acidilactici* (LAB04), *L. brevis* (LAB05), and *L. casei* (LAB06, LAB07, LAB08, LAB09, and LAB10).

Figure 5 presents the FTIR spectra of the microcapsules containing different probiotic strains, as well as pure inulin. Inulin exhibited characteristic polysaccharide bands, most notably a broad and intense band at 3380 cm^−1^, attributed to O–H stretching (hydroxyl groups), associated with the presence of hydroxyl groups and hydrogen-bond formation. This band is also sensitive to moisture, as indicated by the peak at 1645 cm^−1^, which corresponds to the bending vibration of H_2_O molecules and reflects the hygroscopic nature of long-chain inulins [77,78,79]. The high density of hydroxyl groups in inulin is particularly relevant for encapsulation systems, as it enables the establishment of a hydrogen-bonding network capable of stabilizing both the polymeric matrix and biological structures during dehydration processes [80,81].

Other relevant bands include the signal at 2930 cm^−1^, corresponding to C–H stretching (alkyl groups), as well as two distinct bands at 1135 and 1030 cm^−1^. These are attributed to C–O stretching (alcohol group) and asymmetric C–O–C stretching (ether group), respectively, both associated with the saccharide backbone of inulin [79]. The peak at 935 cm^−1^, related to out-of-plane deformation of the –CH_2_ group (methylene group), was also identified and is considered a spectral signature specific to inulin [77,79,82].

In the microencapsulated samples, the main inulin bands were preserved, indicating that their chemical structure remained intact after the encapsulation process. However, subtle changes in band intensity and slight spectral shifts were observed, particularly in the regions corresponding to O–H vibrations (3380 cm^−1^), C–O stretching (1030 cm^−1^), and –CH_2_ deformation (935 cm^−1^). These shifts suggest modifications in the chemical environment of the matrix. Such changes are consistent with the formation of interactions, predominantly hydrogen bonding, between inulin hydroxyl groups and cellular components of the encapsulated probiotic strains, such as membrane polypeptides and exopolysaccharides, reflecting the effective incorporation of microbial biomass into the polymeric matrix during spray-drying microencapsulation. These non-covalent interactions may contribute to a more cohesive matrix–cell interface, potentially reducing molecular mobility within the encapsulating system during dehydration [81,83].

This behavior aligns with the literature, which associates the broadening of the 1030 cm^−1^ band with the overlap of saccharide backbone signals and new hydrophilic interactions between matrix constituents and the encapsulated materials [77,81,82]. Although small variations were observed around 1650 cm^−1^, no defined bands associated with Amide I (1650 cm^−1^) or Amide II (1540 cm^−1^) were detected, suggesting that the amount of encapsulated cellular proteins was below the FTIR detection limit under the experimental conditions used. Nevertheless, the absence of distinct amide bands does not rule out the occurrence of protein–polysaccharide interactions at levels sufficient to influence matrix organization.

### 3.8. X-Ray Diffraction Analysis

**Figure 6:**
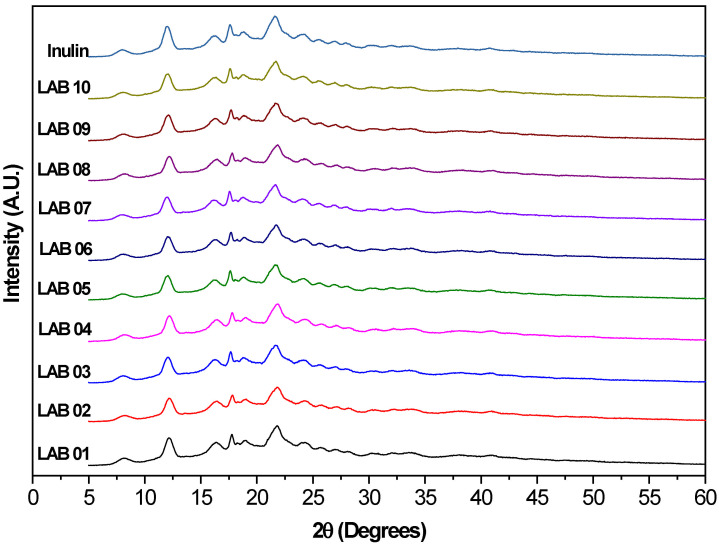
X-ray diffraction (XRD) patterns of probiotic strains microencapsulated with inulin by spray drying: *L. plantarum* (LAB01, LAB02, and LAB03), *P. acidilactici* (LAB04), *L. brevis* (LAB05), and *L. casei* (LAB06, LAB07, LAB08, LAB09, and LAB10), along with the diffraction pattern of pure inulin.

Figure 6 shows the X-ray diffraction patterns of pure inulin and the microcapsules obtained with the different probiotic strains. Pure inulin displayed characteristic and well-defined diffraction peaks, mainly within the 20 range of 10° to 30°, with notable signals at 2θ = 12.2°, 16.4°, 17.7°, 18.8°, 21.3°, 24.5°, and 27.7°, indicating a semicrystalline structural nature [82,84].

The microencapsulated samples (*L. plantarum* LAB01 to *L. casei* LAB10) retained the typical diffraction peaks of inulin, although with reduced intensities and, in some cases, peak broadening. Such changes suggest a partial loss of long-range molecular order within the polymeric matrix after encapsulation, which may be attributed to the interference of microbial biomass in the crystalline organization of the inulin matrix, molecular interactions between the polysaccharides and the cell wall components of the strains, or physical effects associated with the encapsulation technique used [85,86].

Differences in the X-ray diffraction profiles between the microencapsulated samples and pure inulin support the visual observation of reduced peak sharpness and intensity associated with the crystalline phase. This attenuation suggests a decrease in the matrix’s structural order after encapsulation [87]. An increase in the relative amorphous fraction is commonly associated with greater structural flexibility of carbohydrate-based matrices, which may facilitate accommodation of encapsulated materials without compromising the overall integrity of the carrier system. The LAB08 sample stood out by exhibiting more smoothed signals, which may indicate stronger interactions between the *L. casei* strain and the polymeric matrix, or a more pronounced disruption of the crystalline network during processing. Conversely, *L. casei* LAB10 displayed a diffraction pattern more similar to that of pure inulin, suggesting a lower structural impact from the strain or greater compatibility with the matrix. These differences highlight that strain-specific characteristics can influence the extent of structural rearrangement within the encapsulating matrix.

Overall, the results indicate that the inulin matrix retains part of its semicrystalline structure even after encapsulation, which may be technically advantageous. Semicrystalline structures tend to provide enhanced protection to probiotic cells and promote more controlled release in the gastrointestinal tract. At the same time, the coexistence of crystalline and amorphous domains may contribute to balancing structural stability with matrix adaptability, making this parameter relevant in the development of probiotic formulations [81,88].

### 3.9. Thermogravimetric Analysis

**Figure 7:**
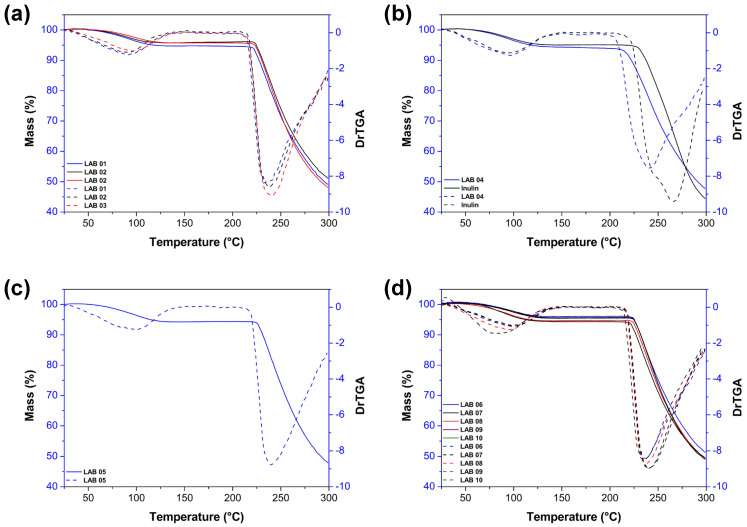
Thermogravimetric analysis (TGA) (solid line) and derivative thermogravimetric analysis (DTGA) (dashed line) of probiotic strains microencapsulated with inulin by spray drying: (**a**) *L. plantarum* (LAB01, LAB02, and LAB03); (**b**) *P. acidilactici* (LAB04) and pure inulin; (**c**) *L. brevis* (LAB05); and (**d**) *L. casei* (LAB06, LAB07, LAB08, LAB09, and LAB10).

Figure 7 illustrates the thermal behavior of the microcapsules containing probiotic strains encapsulated with inulin, evaluated through thermogravimetric analysis (TGA) and its derivative (DTGA). The TGA curves (solid lines) show the mass loss of the samples as a function of temperature, while the DTGA curves (dashed lines) indicate the rate of this mass loss, allowing the identification of temperature ranges associated with major thermal degradation events.

Overall, a thermal profile characterized by two main degradation stages was observed. The first stage, occurring below approximately 120 °C and corresponding to an initial mass loss of 2–5%, is associated with the removal of residual moisture and adsorbed water, as widely reported for microencapsulation systems based on hygroscopic carbohydrates such as inulin [89]. This initial event reflects the water-binding capacity of the matrix, which is influenced by the physicochemical organization of the encapsulating material.

The second stage, marked by an intense degradation event between 220 °C and 270 °C, with substantial mass loss of 35% to 50%, corresponds to the thermal decomposition of the inulin matrix and the cellular components of the probiotic strains, including cell wall proteins and intracellular metabolites [89]. The overlap of these degradation events suggests a close association between the polymeric carrier and the encapsulated biomass within this temperature range.

A comparison among the different formulations revealed that, although subtle variations exist between the strains, all samples exhibited similar thermal behavior, with the onset of significant degradation occurring above 200 °C and DTGA peaks within the 240–260 °C range, indicating the point of greatest degradation rate. These two degradation stages have also been reported in other studies involving inulin-based and probiotic systems, indicating a characteristic thermal pattern for this type of matrix [72,88,90]. The consistency of these thermal profiles suggests that the encapsulation process yields structurally comparable systems, regardless of the probiotic strain employed.

The microcapsules containing *L. brevis* (LAB05) (Figure 7c) showed a DTGA profile with a single, sharp, and well-defined peak, suggesting a more homogeneous structure, possibly related to the smaller average particle size observed for this formulation. In contrast, the formulations with *L. casei* (LAB06 to LAB10) (Figure 7d) exhibited overlapping thermal patterns, reinforcing the reproducibility of the inulin microencapsulation process across different variants of the same species. Such similarities indicate that strain-related differences did not markedly alter the overall thermal stability of the carrier system.

When compared with pure inulin (Figure 7b), the presence of probiotic strains slightly modified the thermal profiles, producing shifts in DTGA peaks and a smoothing of degradation rates, which suggests physicochemical interactions between the functional groups of inulin and cellular components of the probiotics, possibly via hydrogen bonding. These subtle modifications indicate changes in the thermal response of the matrix without altering its fundamental degradation behavior. Although non-covalent, such interactions contribute to enhanced thermal stability of the formulations, in agreement with previous findings for encapsulated systems using prebiotic fibers [81,85].

## 4. Conclusions and Future Perspectives

This study underscores the importance of isolating LAB from foods traditionally consumed by the population, such as artisanal Colonial cheese (ACC), as a sustainable strategy to expand the repertoire of locally adapted strains with potential probiotic and functional applications. Overall, the isolates exhibited desirable traits, including tolerance to simulated gastrointestinal conditions even without microencapsulation, relevant auto-aggregation and cell-surface hydrophobicity, and the absence of key phenotypic virulence-associated activities, supporting their candidacy for further development. At the same time, clear strain-dependent differences were observed across assays, reinforcing the need for a stepwise selection approach. Considering the combined performance and the phenotypic safety screening, *Lactiplantibacillus plantarum* LAB01 and LAB02, as well as *Lacticaseibacillus casei* LAB06 and LAB09 emerges as the most promising candidates to prioritize in the next phase.

Regarding technological viability, spray-dried microencapsulation with inulin resulted in substantial losses in culturability, particularly during storage at 25 °C, whereas refrigerated storage better preserved viability. Importantly, this should not be interpreted as a limitation of inulin itself, since inulin is widely recognized as a protective, prebiotic carbohydrate; rather, the findings indicate that the overall encapsulation and storage conditions (including thermal load, strain physiology, and process parameters) are decisive for stability and must be optimized for each candidate strain. In this context, future work should focus on refining spray-drying settings (e.g., inlet/outlet temperatures and feed conditions), testing protective blends of wall materials that can further mitigate heat and dehydration stress, and validating performance in real food matrices under realistic distribution/storage scenarios—steps that can improve shelf-life while reducing losses and waste, strengthening the sustainability of functional product development.

Finally, because phenotypic screening cannot fully rule out transferable antimicrobial resistance determinants or cryptic virulence factors, the prioritized strains (especially LAB06, LAB09, LAB01 and LAB02) should proceed to genotypic safety assessment (targeted detection of resistance/virulence genes and mobile genetic elements, supported by genome-based analyses). Once genotypic safety is confirmed, in vivo studies should follow to verify safety, persistence, and functional effects, enabling the responsible advancement of these autochthonous LABs toward safe, effective, and more sustainable functional foods.

## Figures and Tables

**Table 1:** MIC and MBC results for LAB isolated from ACC at 14 and 21 days of ripening.

Strains	Antibiotics (µg/mL)													
	Ampicillin		Benzylpenicillin		Streptomycin		Clindamycin		Cephalexin		Metronidazole		Meropenem	
	MIC	MBC	MIC	MBC	MIC	MBC	MIC	MBC	MIC	MBC	MIC	MBC	MIC	MBC
*L. plantarum* LAB01	2	4	4	-	4	64	0.25	0.25	-	-	-	-	0.125	0.125
*L. plantarum* LAB02	0.25	4	16	64	16	-	2	16	64	-	-	-	1	2
*L. plantarum* LAB03	4	8	1	1	32	64	0.125	0.125	32	-	-	-	0.25	4
*P. acidilactici* LAB04	4	-	4	64	64	-	0.5	4	-	-	-	-	2	16
*L. brevis* LAB05	0.5	2	8	32	64	256	2	16	64	-	-	-	2	4
*L. casei* LAB06	0.5	8	1	32	2	64	0.25	2	-	-	-	-	2	-
*L. casei* LAB07	1	8	1	4	16	128	0.25	1	-	-	-	-	4	64
*L. casei* LAB08	8	16	0.25	32	32	32	0.5	2	-	-	-	-	0.5	-
*L. casei* LAB09	2	32	1	2	32	512	0.125	0.5	32	-	-	-	0.25	2
*L. casei* LAB10	4	4	1	32	64	64	0.5	2	-	-	-	-	8	64
*L. plantarum* ATCC8014	4	-	4	64	128	-	0.25	4	-	-	-	-	2	-
*L. casei* BGP93	4	-	4	-	128	1024	0.25	4	-	-	-	-	2	-
***Cutting Point***														
*L. plantarum*	2		0.5		16 *		2		16		4		2	
*L. casei*	4		0.5		64		1		16		4		2	
*L. brevis*	2		0.5		64		1		16		4		2	
*P. acidilactici*	4		0.5		64		1		16		4		2	

MIC: Minimum inhibitory concentration. MBC: Minimum bactericidal concentration. (*): Concentrations ≥ 16 µg/mL were considered resistant. Cut-off values adapted from EUC (2003) [27], EFSA (2012) [28], EUCAST (2021) [29], and Gad et al. (2014) [30].

**Table 2:** Resistance of LAB strains to the stages of simulated in vitro digestion.

Strains	LAB Count (log CFU/g)			Survival Rate (%)	
	Initial	Stomach	Intestine	Stomach	Intestine
*L. plantarum* LAB01	8.53 ± 0.02 ^abA^	8.33 ± 0.10 ^aA^	7.65 ± 0.10 ^aB^	97.60 ± 1.39 ^abA^	89.69 ± 1.01 ^aB^
*L. plantarum* LAB02	8.65 ± 0.03 ^aA^	8.12 ± 0.12 ^abcB^	7.77 ± 0.02 ^aC^	93.87 ± 1.03 ^abA^	89.76 ± 0.47 ^aB^
*L. plantarum* LAB03	8.08 ± 0.40 ^abA^	8.10 ± 0.14 ^abcA^	7.69 ± 0.55 ^aA^	100.37 ± 3.19 ^aA^	95.12 ± 2.13 ^aA^
*P. acidilactici* LAB04	8.44 ± 0.03 ^abA^	7.46 ± 0.12 ^bcB^	7.14 ± 0.09 ^aB^	88.43 ± 1.72 ^bA^	84.63 ± 1.38 ^aA^
*L. brevis* LAB05	8.77 ± 0.08 ^aA^	8.31 ± 0.11 ^abB^	7.95 ± 0.06 ^aB^	94.73 ± 0.30 ^abA^	90.71 ± 0.16 ^aB^
*L. casei* LAB06	8.57 ± 0.27 ^abA^	8.06 ± 0.31 ^abcA^	7.58 ± 0.07 ^aA^	94.08 ± 0.66 ^abA^	88.53 ± 1.90 ^aA^
*L. casei* LAB07	8.49 ± 0.13 ^abA^	7.74 ± 0.11 ^abcB^	7.46 ± 0.09 ^aB^	91.22 ± 2.70 ^abA^	87.95 ± 0.23 ^aA^
*L. casei* LAB08	7.89 ± 0.07 ^bA^	7.36 ± 0.02 ^cB^	7.02 ± 0.13 ^aB^	93.22 ± 1.14 ^abA^	88.90 ± 0.91 ^aA^
*L. casei* LAB09	8.27 ± 0.13 ^abA^	7.90 ± 0.43 ^abcA^	7.45 ± 0.39 ^aA^	95.48 ± 3.73 ^abA^	90.20 ± 6.19 ^aA^
*L. casei* LAB10	8.32 ± 0.29 ^abA^	7.45 ± 0.31 ^cA^	7.37 ± 0.28 ^aA^	89.72 ± 6.84 ^abA^	88.76 ± 6.39 ^aA^

Results expressed as mean ± standard deviation. ^a–c^ Different lowercase letters indicate significant differences among strains within the same digestion stage (*p* < 0.05). ^A–C^ Different uppercase letters indicate significant differences among digestion stages for the same bacterial strain (*p* < 0.05).

**Table 3:** Auto-aggregation and hydrophobicity results of potentially probiotic LAB isolated from ACC.

Strains	Auto-Aggregation (%)			Hydrophobicity (%)
	3 h	6 h	24 h	
*L. plantarum* LAB 01	31.62 ± 4.24 ^aB^	38.96 ± 6.21 ^aB^	73.56 ± 6.24 ^aA^	76.69 ± 4.68 ^d^
*L. plantarum* LAB 02	33.08 ± 1.36 ^aB^	42.80 ± 0.66 ^aB^	62.72 ± 4.57 ^aA^	87.41 ± 3.98 ^b^
*L. plantarum* LAB 03	32.47 ± 2.94 ^aB^	37.76 ± 5.06 ^aB^	73.18 ± 6.55 ^aA^	56.06 ± 2.25 ^e^
*P. acidilactici* LAB 04	30.64 ± 3.17 ^aB^	32.44 ± 6.85 ^aB^	74.33 ± 3.49 ^aA^	45.70 ± 4.40 ^f^
*L. brevis* LAB 05	29.43 ± 0.18 ^aB^	42.96 ± 2.44 ^aB^	78.44 ± 8.73 ^aA^	99.67 ± 0.09 ^a^
*L. casei* LAB 06	23.53 ± 0.98 ^aB^	28.41 ± 1.03 ^aB^	68.84 ± 8.83 ^aA^	95.38 ± 0.61 ^ab^
*L. casei* LAB 07	22.08 ± 5.14 ^aB^	39.68 ± 0.45 ^aAB^	65.69 ± 11.15 ^aA^	97.72 ± 0.20 ^a^
*L. casei* LAB 08	30.72 ± 11.21 ^aA^	34.10 ± 16.00 ^aA^	78.76 ± 4.12 ^aA^	78.66 ± 0.63 ^cd^
*L. casei* LAB 09	36.94 ± 0.29 ^aB^	35.83 ± 3.37 ^aB^	77.82 ± 3.09 ^aA^	53.37 ± 0.87 ^ef^
*L. casei* LAB 10	20.29 ± 7.38 ^aB^	27.50 ± 6.66 ^aB^	75.04 ± 6.82 ^aA^	93.16 ± 0.74 ^ab^

Results are expressed as mean ± standard deviation. ^a–f^ Different lowercase letters indicate significant differences among strains at the same auto-aggregation time point (*p* < 0.05). ^A,B^ Different uppercase letters indicate significant differences among auto-aggregation times for the same bacterial strain *(p* < 0.05).

## Data Availability

The original contributions presented in this study are included in the article. Further inquiries can be directed to the corresponding author.
